# Sunitinib-induced cardiac hypertrophy and the endothelin axis

**DOI:** 10.7150/thno.49837

**Published:** 2021-02-06

**Authors:** Joevin Sourdon, Caterina Facchin, Anaïs Certain, Thomas Viel, Blaise Robin, Franck Lager, Carmen Marchiol, Daniel Balvay, Thulaciga Yoganathan, Judith Favier, Pierre-Louis Tharaux, Neeraj Dhaun, Gilles Renault, Bertrand Tavitian

**Affiliations:** 1 Université de Paris, PARCC, INSERM, F-75015 Paris.; 2 Aix-Marseille Université, CNRS, CRMBM, UMR 7339, Marseille, France.; 3 INSERM U1016, Institut Cochin, Université de Paris, Paris 75014, France.; 4 Équipe labellisée par la Ligue contre le Cancer.; 5 Équipe labellisée par la Fondation pour la Recherche Médicale.; 6 University/British Heart Foundation Centre of Research Excellence, The Queen's Medical Research Institute, University of Edinburgh, United Kingdom.; 7 Service de radiologie, AP-HP, Hôpital européen Georges Pompidou, F-75015 Paris.

**Keywords:** cardiotoxicity, sunitinib, endothelin, cardiac hypertrophy, metabolic imaging.

## Abstract

Anti-angiogenics drugs in clinical use for cancer treatment induce cardiotoxic side effects. The endothelin axis is involved in hypertension and cardiac remodelling, and addition of an endothelin receptor antagonist to the anti-angiogenic sunitinib was shown to reduce cardiotoxicity of sunitinib in mice. Here, we explored further the antidote effect of the endothelin receptor antagonist macitentan in sunitinib-treated animals on cardiac remodeling.

**Methods:** Tumor-bearing mice treated *per os* daily by sunitinib or vehicle were imaged before and after 1, 3 and 6 weeks of treatment by positron emission tomography using [^18^F]fluorodeoxyglucose and by echocardiography. Non-tumor-bearing animals were randomly assigned to be treated *per os* daily by vehicle or sunitinib or macitentan or sunitinib+macitentan, and imaged by echocardiography after 5 weeks. Hearts were harvested for histology and molecular analysis at the end of *in vivo* exploration.

**Results:** Sunitinib treatment increases left ventricular mass and ejection fraction and induces cardiac fibrosis. Sunitinib also induces an early increase in cardiac uptake of [^18^F]fluorodeoxyglucose, which is significantly correlated with increased left ventricular mass at the end of treatment. Co-administration of macitentan prevents sunitinib-induced hypertension, increase in ejection fraction and cardiac fibrosis, but fails to prevent increase of the left ventricular mass.

**Conclusion:** Early metabolic changes predict sunitinib-induced cardiac remodeling. Endothelin blockade can prevent some but not all cardiotoxic side-effects of sunitinib, in particular left ventricle hypertrophy that appears to be induced by sunitinib through an endothelin-independent mechanism.

## Introduction

Cardio-oncology is an interdisciplinary field aiming to better understand and explore methods in order to limit the cardiotoxicity of cancer therapies. We 1 and others 2 previously showed that the anti-angiogenic drug sunitinib induces cardiac fibrosis and impairs myocardial metabolism and heart dysfunction. Remarkably, the cardiotoxic effects of sunitinib were prevented by concurrent administration of the endothelin (ET) antagonist macitentan 1, suggesting that regulation of the endothelin axis may prevent cardiac side effects of anti-angiogenics, with potential clinical interest.

Hypertension is a common side effect of anti-angiogenic treatment 3. In one study, approximately 50% of patients had a significant increase of blood pressure during their first course of sunitinib treatment 4. Another study reported that approximately 80% of patients developed hypertension after two courses of sorafenib 5. An array of research indicates that up-regulation of the ET pathway during sunitinib treatment is responsible for arterial hypertension 6. Kappers *et al.* reported high serum concentrations of ET-1 associated with hypertension in 15 patients treated with sunitinib 6, and showed that ET inhibition prevented sunitinib-induced hypertension in swine 7. Both hypertension and endothelin lead to myocardial remodeling 8-10. Therefore, an array of arguments is in favor of adding an anti-endothelin drug to anti-angiogenic therapy in order to prevent long-term cardiotoxicity. Following the road towards clinical translation, we tested in mice models with and without tumors the effect of sunitinib-induced cardiotoxicity with a special focus on the prevention of long-term cardiac hypertrophy by macitentan.

## Materials and Methods

Animal experiments complied to French and European regulations and were approved by the Paris Descartes Animal Experimentation Ethics Committee (CEEA34) registered under reference #12859, #13042 and 15-045 and performed by certified staff following the French law of animal experimentation n°2013-118. NMR nude (nu/nu) female (Janvier Labs, France) and C57BL/6 mice (Janvier Labs, France) 6 weeks of age were maintained in controlled temperature (24 °C) and relative humidity (50%) on a 12/12-light/dark cycle and were fed *ad libitum*. Sunitinib malate (Cliniscience, A10880-500) was dissolved at 10 mg/mL in DMSO/PBS (1:4). Sunitinib was administered daily by oral gavage at a dose of 50 mg/kg body weight and macitentan by oral gavage in a dose of 20 mg/mL. In the sunitinib+macitentan group, macitentan a clinically-available mixed ET_A_ and ET_B_ receptor antagonist, was added to the gavage solution with the volume maintained at 200 μL.

**Figure 1:**
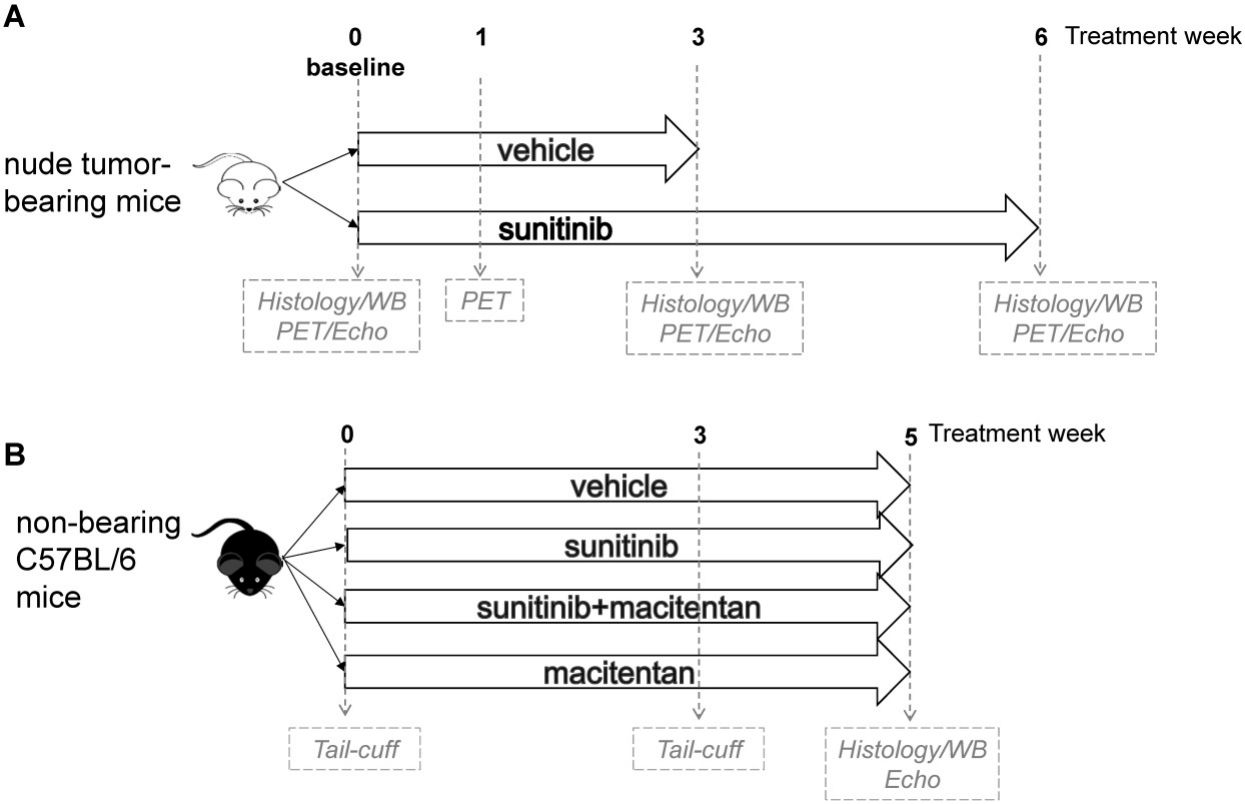
** Study design: (A)** A total of 23 tumor bearing nude mice were studied. 16 mice were imaged by PET and echography at baseline, week 1 and week 3, either under sunitinib (n = 8) or vehicle (n = 8) treatment and after 6 weeks of sunitinib treatment. Heart samples were collected at baseline (n = 4), week 3 (vehicle: n = 8; sunitinib: n = 3) and week 6 (n = 8). **(B)** non-tumor bearing mice (n = 28) were imaged by echography after 5 weeks of treatment with vehicle (n = 6) or sunitinib (n = 8) or macitentan (n = 7) or sunitinib+macitentan (n = 7). Hearts were collected at the last PET/echo scan. Echo: echocardiography; PET: Positron emission tomography; WB: western blot.

In a first panel, the study design followed a standard protocol of mouse oncology studies with PET in fasted, tumor-bearing, nude mice for monitoring of short-term (as previously studied 1) and long-term response to sunitinib. Allografts of tumors obtained from immortalized mouse cromaffin cells (imCC) carrying a homozygous knockout of the *Sdhb* gene (*Sdhb*^-/-^) were propagated in the neck fat pad of NMR nude female mice (n = 23). This artificial paraganglioma tumor model does not secrete catecholamines 11, therefore excluding a confounding action of these hormones on cardiac function. Mice were used to assess early metabolic changes in a standard mouse oncology positron emission tomography (PET) protocol. Sunitinib was administered during 6 weeks (n = 8) and vehicle solution along 3 weeks (n = 8). At that time the *Sdhb^-/-^* tumor volume exceeded the UKCCCR recommended limit 12 and mice were sacrificed. PET scan was performed before and after 1 and 3 weeks of treatment; cardiac echography (echo) was performed before and after 3 weeks of sunitinib malate (n = 8) or vehicle (n = 8), and finally animals were monitored by imaging after 6 weeks treatment (**Figure 1A**). For histology and western-blotting, tumor-bearing mice were sacrificed at baseline (n = 4), after 3 weeks (n = 3 for sunitinib, n = 8 for vehicle) and 6 weeks of sunitinib treatment (n = 8).

Immune competent mice were used in a second series of experiments to check whether blockade of endothelin pathway could prevent sunitinib-induced LV mass. C57BL/6 female mice were separated in four groups: sunitinib (n = 8), sunitinib+macitentan (n = 7), vehicle (n = 6), and macitentan (n = 7). All mice were followed for arterial pressure measurement during three weeks and a cardiac echography was realized after 5 weeks of treatment (**Figure 1B**). Non-tumor-bearing mice were sacrificed after 5 weeks of treatment.

### Cardiac Positron Emission Tomography (PET)

Early metabolic changes were assessed by PET scan with 2'-deoxy-2'-[^18^F]fluoro-D-glucose (^18^F-FDG). PET sessions were realized in a PET-CT scanner (nanoScan PET-CT; Mediso, Hungary) using 10 MBq of ^18^F-FDG (Advanced Applied Applications, France) as already published 1. Quickly, tumor-bearing mice were fasted overnight, anesthetized (2 ± 0.5% isoflurane in air), weighed and glycaemia was measured in blood drawn from the caudal artery. ^18^F-FDG accumulation was quantified as mean SUV between 45 and 60 min post-injection in 3D volumes-of-interest (VOI) delineated semi-automatically by iso-contours at 45% threshold of maximal value in the myocardium on PET/CT fusion slices using the PMOD software package (PMOD Technologies Ltd, Zürich, Switzerland).

### Echocardiography

Conventional echocardiography was performed in anesthetized mice using a Vevo 2100 high resolution ultrasound device (Visualsonics, Toronto, Canada) with a 40 MHz probe (MS-550). Mice were anesthetized with 3% isoflurane in air for induction and maintained with 1.5%. Mice were depilated in the thoracic region and placed in the supine position on a dedicated heating platform, allowing monitoring of ECG, temperature and respiratory frequency. All acquisitions were performed within body temperature limits 36-37.5 °C. Parasternal long axis views were recorded and 3 consecutive measurements in M-mode were drawn to determine left ventricular internal diameter (LVID), interventricular septum (IVS), and left ventricular posterior wall thickness (LVPW) in both end diastole (d) and end systole (s)**.** LV fractional shortening (M-Mode) was calculated with the following equation: 100 * ((LVID;d - LVID;s) / LVID;d), LV mass was calculated as: (1.053 x ((LVID;d + LVPW;d + IVS;d)3 - LVID;d3)) x 0.8. Ejection Fraction (B-Mode) was calculated as [(telediastolic volume - telesystolic volume) / telediastolic volume].

### Arterial pressure Measurement

Arterial pressure was measured at baseline and after 3 weeks of treatment (vehicle, sunitinib, sunitinib+macitentan and macitentan) in a VISITECH-System (BP2000, BP2 - 002250).

### Assessment of cardiac fibrosis

Paraffin hearts sections were incubated with Picrosirius red (VWR) 0.1% in picric acid (Sigma) in a Leica ST5020 automatic strainer during 30 min and dehydrated in ethanol and xylene. Whole sections were observed at a magnification 20x using a Nanozoomer HT 2.0 (Hamamatsu) and fibrosis was quantified using FIBER, Matlab®-based software.

### Western blotting

Twenty micrograms of heart lysate were loaded onto a 10% SDS-PAGE gels (mini-protean TGX gels, BioRad) and transferred to nitrocellulose membranes. The membranes were blocked and incubated with the following primary antibodies: p-ERK (rabbit anti-mouse, CS 9101S, 1:1000), ERK (rabbit anti-mouse, 9102S, 1:1000) and GAPDH (CS2118S, 1:1000). Membranes were then incubated with anti-rabbit secondary antibodies (goat anti-rabbit AB217673), imaged with LI-COR Odyssey imaging system and quantified using Image Studio Lite 2.

### Statistics

Data are expressed as mean ± SEM. Analysis was performed with Graphpad Prism 7.00 (GraphPad Software, San Diego, California, USA). One-way ANOVA was used to compare four data sets at one time point, and two-way ANOVA was used to compare four groups followed across time with Bonferroni correction. Unpaired and paired *t*-tests were used to compare two data sets after d'Agostino and Pearson's normality test. *p* values were considered significant when *p* < 0.05.

## Results

A first leg of study was designed to monitor longitudinally, using PET and echocardiography once per week, the cardiovascular side-effects of sunitinib in tumor-bearing immune deficient mice (**Figure 1A**). Rapid tumor growth (**Figure S1**) in the vehicle-treated group was observed during 3 weeks; after what the mice had to be euthanized (UKCCCR recommendations), while in the sunitinib-treated group tumors grew slower and were monitored over 6 weeks. To homogenize treatment durations, to account for the role of innate immunity during the cardiac response to antitumor therapy and to investigate the mechanism underlying sunitinib-induced increased LV mass, C57BL/6 immunocompetent mice received vehicle or sunitinib or macitentan or sunitinib+macitentan treatments of similar durations. Mean arterial pressure, cardiac imaging and image-derived cardiac parameters were observed during 5 weeks (**Figure 1B**). All hearts were collected for histology and Western blot analysis after completion of treatments.

### Sunitinib induces cardiac remodeling

**Figure 2:**
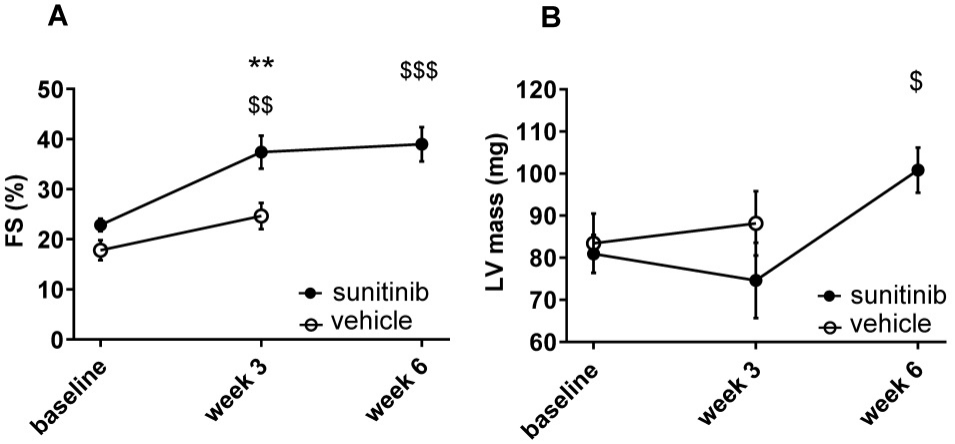
** Sunitinib induces cardiac dysfunction: (A)** Left ventricular fractional shortening measured at baseline, week 3 and week 6 for vehicle (open circles) and sunitinib-treated (filled circles) tumor bearing nude mice. **(B)** LV mass measured at baseline, week 3 and week 6 for vehicle (open circles) and sunitinib-treated (filled circles) tumor bearing nude mice. n = 8 per group. Data are expressed as mean ± SEM, ^**^ *p* < 0.01 compared to vehicle; ^$^ *p* < 0.05, ^$$^ *p* < 0.01 and ^$$$^ *p* < 0.001 compared to baseline for sunitinib group. FS: Fractional shortening; LV: left ventricle.

**Figure 3:**
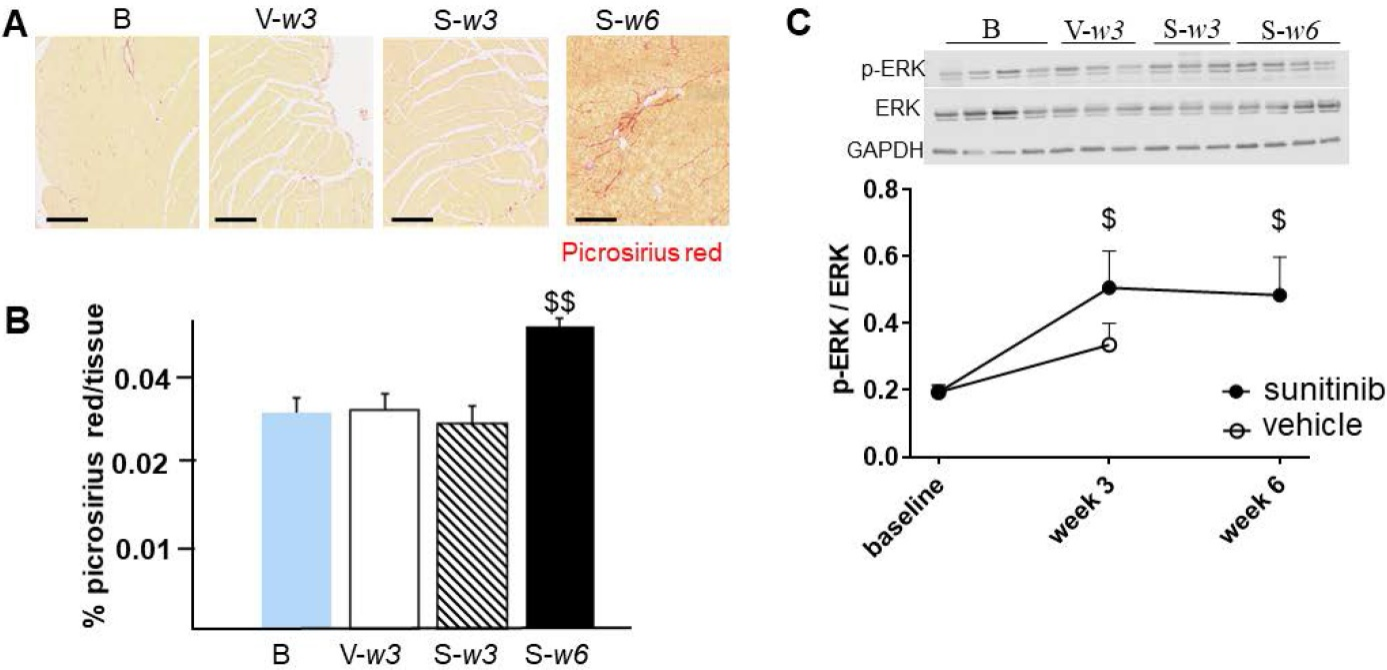
** Sunitinib induces cardiac remodeling: (A)** Representative sections of myocardium stained for fibrosis (Picrosirius red) from hearts of non-treated mice **(B)** and mice treated with vehicle at week 3 (V-w3), or sunitinib at week 3 (S-w3) and week 6 (s-w6). Scale bar: 200 µm. **(B)** Quantification of fibrosis (normalized by tissue area) in tumor bearing nude mice non treated (blue, n = 4), treated with vehicle at week 3 (white, n = 8) or sunitinib at week 3 (striped, n = 3) and week 6 (black, n = 8). **(C)** Representative blots and their associated quantification for p-ERK and ERK (normalized by GAPDH) of tumor bearing nude mice treated with vehicle (open circles) or sunitinib (filled circles). Data expressed as mean ± SEM. ^$^ *p* < 0.05 and ^$$^ *p* < 0.01 compared to baseline for sunitinib group. ERK: Extracellular signal-regulated kinases.

Fractional shortening increased at week 3 and week 6 of sunitinib treatment (**Figure 2A**). In addition, LV mass was significantly increased after 6 weeks of sunitinib compared with baseline (75 ± 8.9 mg vs. 101 ± 5.3 mg, *p* < 0.05) (**Figure 2B**). Sunitinib treatment also induced higher cardiac fibrosis at week 6 in comparison with baseline (*p* < 0.01) (**Figure 3A-B**) and increased phosphorylation (*p* < 0.05) of Extracellular signal-Regulated Kinases (ERK) pathway when compared with non-treated mice (**Figure 3C**).

### Sunitinib enhances cardiac glycolytic metabolism at W1

**Figure 4:**
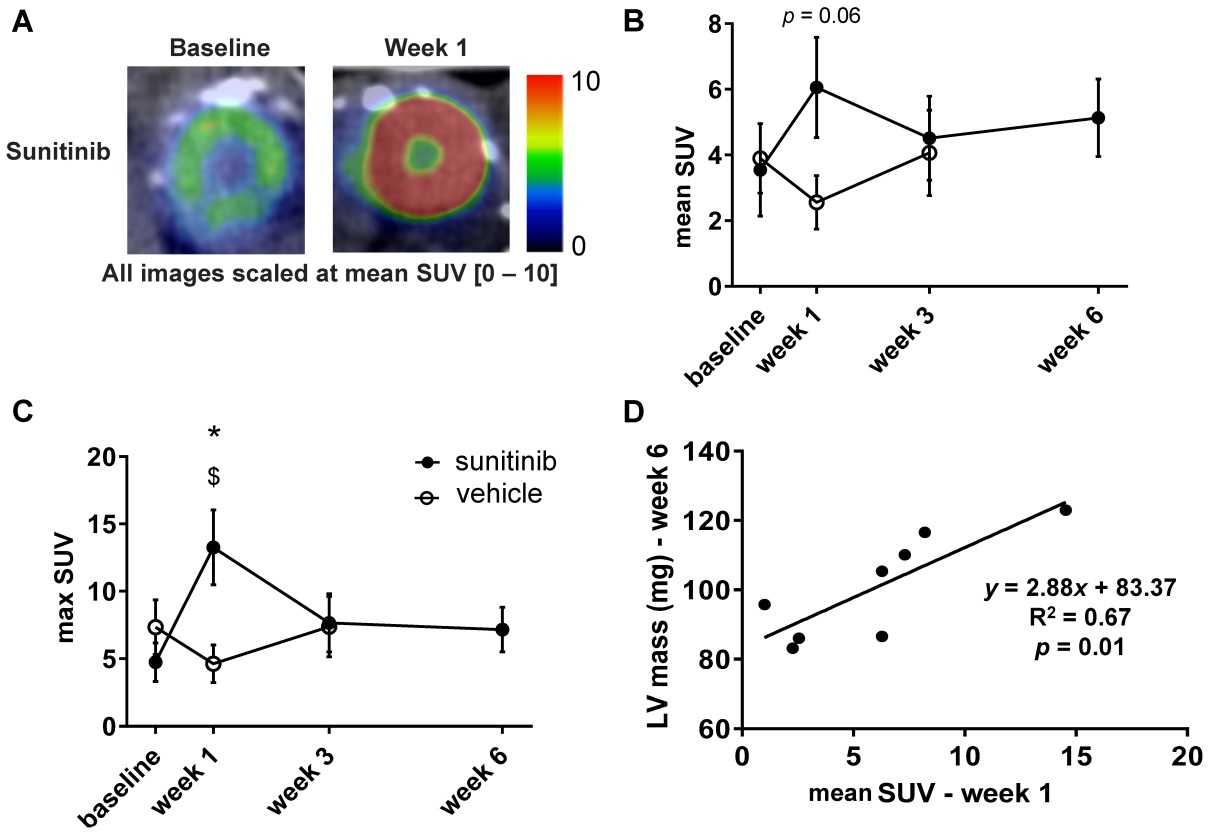
** Sunitinib enhances cardiac glycolytic metabolism at W1: (A)** Example of PET scan images representing cardiac short axis view of FDG-SUV at baseline (left) and after one week (right) of sunitinib treatment. **(B)** Mean SUV measured at baseline, week 1, week 3 and week 6 for vehicle (open circles) and sunitinib-treated (filled circles) tumor bearing nude mice. **(C)** Max SUV measured at baseline, week 1, week 3 and week 6 for vehicle (open circles) and sunitinib-treated (filled circles) tumor bearing nude mice. **(D)** Pearson correlation between mean SUV at W1 and LV mass at W6 in sunitinib group. Each dot represents one mouse. Linear regression equation and coefficient of correlation (R) with R p-value are shown. n = 8 per group. Data are expressed as mean ± SEM, ^*^ *p* < 0.05 compared to vehicle; ^$^ *p* < 0.05 compared to baseline for sunitinib group; LV: left ventricle; SUV: standard uptake values.

One week of treatment with sunitinib increased cardiac ^18^F-FDG uptake by nearly 40% (**Figure 4A-B**). The max SUV at week 1 also augmented by 60% in comparison with vehicle-treated animals (*p* < 0.05) (**Figure 4C**). The mean SUV increase from baseline to week 1 was correlated with the LV mass assessed at week 6 (*p* < 0.01) (**Figure 4D**), suggesting a link between early cardiac metabolic changes and delayed hypertrophic heart remodeling.

### Macitentan prevents sunitinib-induced hypertension

**Figure 5:**
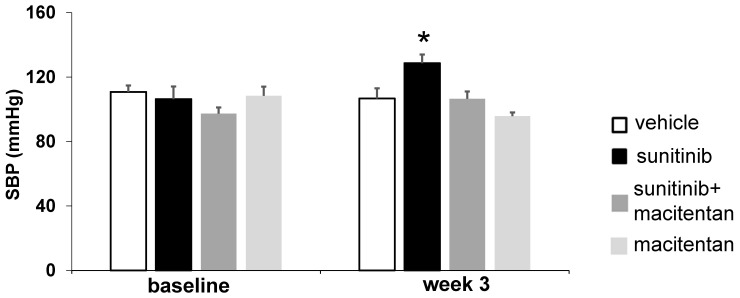
** Macitentan prevents sunitinib-induced hypertension:** Arterial pressure measured at baseline and after 3 weeks of vehicle (white, n = 6), sunitinib (black, n = 8), sunitinib+macitentan (dark grey, n = 7) and macitentan (grey, n = 7) treatment in non-tumor bearing mice. Data expressed in mean ± SEM. * *p* < 0.05 compared to other groups. SBP: systolic blood pressure. SBP: systolic blood pressure.

Three weeks of sunitinib treatment increased systolic blood pressure by 20% compared with baseline (106 ± 7.8 mmHg at baseline vs. 129 ± 5.4 mmHg at week 3, *p* < 0.05) (**Figure 5**). Co-administration of macitentan to sunitinib prevented the rise in systolic blood pressure in 7 mice out of 8, and as a result the mean systolic blood pressure in the sunitinib+macitentan group (107 ± 4.5 mmHg) was not different from baseline (**Figure 5**).

### Macitentan prevents cardiac dysfunction and cardiac fibrosis

**Figure 6:**
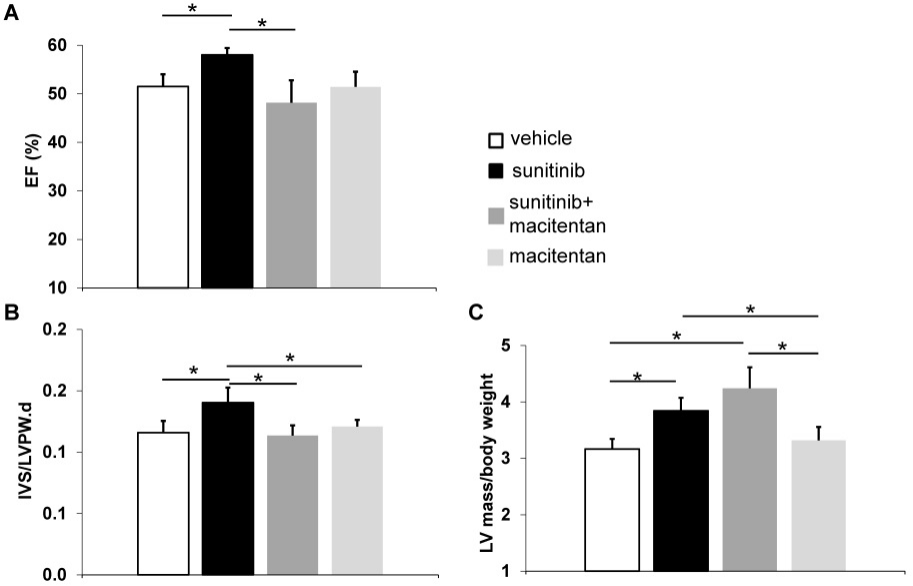
** Macitentan prevents cardiac dysfunction: (A)** Left ventricular ejection fraction measured after 5 weeks of vehicle (white), sunitinib (black), sunitinib+macitentan (dark grey) and macitentan (grey) treatment. **(B)** Interventricular septum/left ventricular posterior wall ratio measured after 5 weeks of vehicle (white), sunitinib (black), sunitinib+macitentan (dark grey) and macitentan (grey) treatment. **(C)** Left ventricular mass measured after 5 weeks of vehicle (white), sunitinib (black), sunitinib+macitentan (dark grey) and macitentan (grey) treatment. n = 6-8 per group. Data expressed in mean ± SEM. * *p* < 0.05 between two related samples. EF: ejection fraction; IVS: Interventricular septum; LVPW: Left ventricular posterior wall.

**Figure 7:**
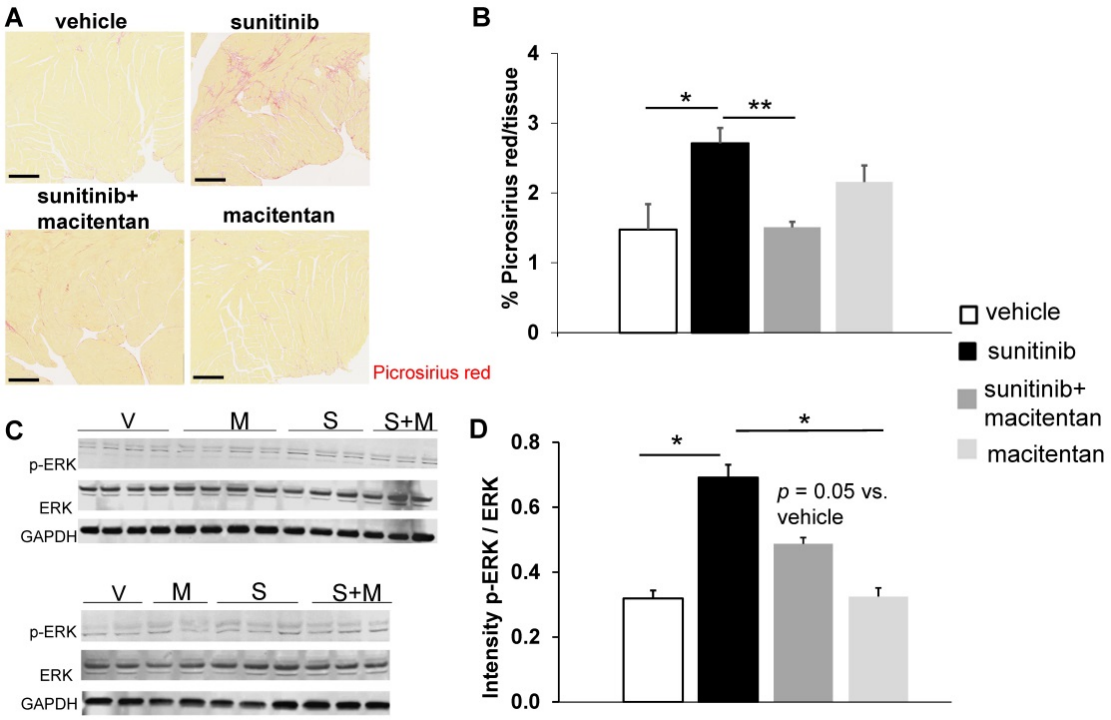
** Macitentan prevents sunitinib-induced cardiac fibrosis: (A)** Representative sections of myocardium stained for fibrosis (Picrosirius red) from hearts of mice treated with vehicle (upper left), sunitinib (upper right), sunitinib+macitentan (bottom left) and macitentan (bottom right). Scale bar: 200 µm. **(B)** Quantification of fibrosis (normalized by tissue area) in C57Bl/6, non-tumor bearing mice treated with vehicle (white, n = 6), sunitinib (black, n = 8), sunitinib+macitentan (dark grey, n = 7) and macitentan (grey, n = 7) treatment. **(C)** Representative blots and their associated quantification **(D)** for p-ERK and ERK (normalized by GAPDH) in C57Bl/6 mice treated with vehicle (white, n = 6), sunitinib (black, n = 6), sunitinib+macitentan (dark grey; n = 6) and macitentan (grey, n = 6). Data expressed in mean ± SEM. * *p* < 0.05; ** *p* < 0.01 between two related samples. ERK: Extracellular signal-regulated kinases.

Ejection fraction (EF) rose to 58 ± 1.4% in the sunitinib-treated group at week 5 (*p* < 0.05), compared to 51 ± 2.5% in control animals. Animals receiving macitentan and sunitinib had an EF of 46 ± 5.0% (**Figure 6A**). Macitentan prevented sunitinib-induced fibrosis as shown by histology (**Figure 7A-B**).

### Macitentan fails to prevent sunitinib-induced cardiac hypertrophy

At week 5, sunitinib-treated animals had a higher inter ventricular septum (IVS) to LVPW ratio in comparison to vehicle-treated animals (*p* < 0.05). This effect was prevented by the addition of macitentan (**Figure 6B**). In contrast, the ratio of LV mass/body weight was increased significantly (*p* < 0.05) in both the sunitinib (3.8 ± 0.65) and sunitinib+macitentan (4.3 ± 0.39) groups, in comparison to the vehicle and the macitentan groups (3.2 ± 0.18 and 3.3 ± 0.63, respectively) (**Figure 6C**). Increased LV mass at week 5 in sunitinib and sunitinib+macitentan treated animals was associated with a higher phosphorylation of ERK pathway in those groups when compared with vehicle-treated mice (**Figure 7C-D**).

## Discussion

Cardiotoxicity is a concern for the clinical use of antiangiogenics. Here we show that sunitinib induces cardiac hypertrophy and that early metabolic changes assessed by *in vivo* FDG-PET imaging after 1 week of treatment can predict delayed cardiac remodeling. Sunitinib- associated cardiac remodeling is shown by elevation of LV mass (**Figures 2-6**) and accumulation of collagen fibers triggered by activation of hypertrophic molecular pathways (ERK).

The activation of the endothelin axis by sunitinib may explain its cardiac side effects. Indeed, ET-1 induces fibrosis and myocardial hypertrophy 9, 10 partially through upregulation of transglutaminase 2 (TG2) 13. In mice, increased expression of TG2 has been found responsible for diastolic dysfunction, cardiac chamber dilation as well as remodeling of extracellular matrix 14. Furthermore, as TG2 participates to cardiac remodeling 15, 16 and is activated by hyper glycolysis 17, our observation that sunitinib induces early abnormal higher glucose uptake in nude mice after one week of treatment suggests that this could activate the TG2 pathway and trigger cardiac remodeling.

As expected, ET-antagonism by macitentan prevents sunitinib-induced pressure overload, prevents fibrosis, prevents sunitinib-induced hypertension, and maintains EF at physiological levels. Our results confirm previous reports relating that blockade of the ET axis protects against pressure-overload 7, fibrosis 18, 19 and myocardial metabolic deregulation 1. In addition, we speculate that the blockade of ET axis may protect against activation of TG2 13 and against vasoconstriction 20. Endothelin antagonists inhibit cardiac hypertrophy 18, 19, 21 and are used to treat right ventricle remodeling in pulmonary hypertension 22, 23. Therefore, it is quite surprising that adding macitentan to a sunitinib regimen has no effect on long-term delayed cardiac hypertrophy and fails to protect against myocardial enlargement. The partial protection of sunitinib-induced cardiotoxicity by macitentan is however in agreement with our previous proteomics analysis 1 summarized in Table 1 (accessible in Proteomics Identification Database with the dataset identifier PXD006888). These results suggest that other signaling pathways than the endothelin axis are involved during sunitinib-induced myocardial remodeling.

Sunitinib inhibits vascular endothelial growth factor receptor (VEGFR) and platelet-derived growth factor (PDGFR). Since the activation of VEGFR through different stimuli is directly responsible for cardiac hypertrophy 24, it is unlikely that its inhibition is responsible for the myocardial remodeling that we observed here. The other possibility is that PDGFR inhibition is, at least in part, responsible for cardiac remodeling induced by sunitinib. In support of this hypothesis, Chintalgattu *et al.* showed that specific inactivation of PDGFR in cardiomyocytes induced LV hypertrophy and fibrosis after 14 days of thoracic aortic constriction 25. The same authors also observed a significant inhibition of AKT, suggesting that the impairment of AKT regulation of metabolism could be involved in cardiac impairment. We show here that sunitinib increases the Mitogen-Activated Protein Kinases (MAPK/ERK) pathway, a key player in myocardial hypertrophy 26, 27, in agreement with a previous report in isolated cardiomyocytes 28. In addition, proteomics analyses (**Figure S2 and Table S1**) show that the higher expression of MAPK/ERK is involved in hypertrophic pathways, such as: Role of NFAT in Cardiac Hypertrophy 29, Protein Kinase A Signaling 30 and NRF2-mediated Oxidative Stress Response 31. The increase of MAPK/ERK could be the consequence of sunitinib-induced inhibition of cardiac AMP-activated protein kinase (AMPK) 32, which is an anti-hypertrophy protein that inhibits MAPK/ERK pathway 33 and which is involved in the regulation of cardiac metabolism 34. In addition to the inhibition of AMPK 32 and AKT 25 and to the impaired cardiac glucose metabolism by sunitinib, this antiangiogenic is also known to induce mitochondrial dysfunction and anaerobic metabolism 1, 2. Hence, there appears to be a link between the impairment of local metabolism and remodeling of the heart. Metabolic imbalance with mitochondrial dysfunction, such as mismatch of oxidative metabolism and reduced ATP production, generate reactive oxygen species (ROS) in mitochondria 35, 36. ROS generation has been documented in the sunitinib-treated myocardium 37, 38. ROS activate the MAPK/ERK pathway 39. Their contribution to pathologic cardiac hypertrophy is well-established 40, for instance in the concentric hypertrophy 41, in diabetic cardiomyopathies 42, 43, in hypertrophic heart disease 44, 45 and in heart failure with preserved ejection fraction (HFpEF) 46-48. The present study demonstrates that sunitinib induces pressure overload, increases LV mass and the IVS/LVPW ratio, induces fibrosis and increases EF, all characteristics of concentric hypertrophy 49. We thus speculate that, in sunitinib-treated animals, macitentan prevents cardiac concentric hypertrophy but has no effect on eccentric hypertrophy, characterized by the enlargement of chamber volume and typical of dilated cardiomyopathies 50, we observed in the sunitinib+macitentan group.

The present results also confirm that sunitinib induces early increase in ^18^F-FDG uptake after 1 week of treatment 1, 2. The early metabolic abnormality correlates specifically with the increase in LV mass at 6 weeks of sunitinib treatment. This result underlines the important link between early cardiac metabolic deregulation and the late stage of cardiac hypertrophy implying an overactivation of the MAPK/ERK pathway. Similar results have been obtained in a mouse model of pressure-overload in which early SUV-^18^F-FDG increase was associated with late cardiac hypertrophy 51. Since several lines of evidence suggest that early metabolic changes in glycolytic metabolism could be predictive of late-heart remodeling 51, 52, using whole-body ^18^F-FDG-PET scans performed in cancer patients may turn clinically useful to evaluate the cardiotoxicity of sunitinib.

## Conclusion

Our results highlight the contribution of multi-organ/multi-modality *in vivo* imaging for a theranostics approach as proposed earlier 53, 54. Such approaches demonstrate that sunitinib-induced cardiotoxicity has several origins, involving *a minima* pressure-overload, the endothelin axis, and a third mechanism or pathway that remains to be determined for late cardiac hypertrophy.

## Supplementary Material

Supplementary figures and tables.

## Figures and Tables

**Table 1:** Major changes in cardiac remodeling related pathways.

		Sunitinib vs. vehicle	Sunitinib + macitentan vs. vehicle group	Sunitinib vs. both sunitinib + macitentan & vehicle groups
Categories	Diseases or Functions Annotation	*p*-Value	*p*-Value	*p*-Value
Cardiovascular Disease	Hypertension	0.002 *up-regulation*	n.s *no difference*	0.009 *up-regulation*
Organismal Injury and Abnormalities	Fibrosis	0.002 *up-regulation*	n.s *no difference*	0.003 *up-regulation*
Cardiovascular Disease, Developmental Disorder, Organismal Injury and Abnormalities	Hypertrophy	< 0.001 *up-regulation*	< 0.001 *up-regulation*	0.005 *up-regulation*
Canonical pathways	Role of NFAT in Cardiac Hypertrophy	0.05 *up-regulation*	n.s *no difference*	n.s *no difference*
Canonical pathways	Protein Kinase A Signaling	0.05 *up-regulation*	0.01 *up-regulation*	n.s *no difference*
Canonical pathways	NRF2-mediated Oxidative Stress Response	0.003 *up-regulation*	0.008 *up-regulation*	n.s *no difference*

Table showing the most significantly pathways perturbed by sunitinib in mouse myocardium independently of endothelin axis. n = 6 per group. n.s: non-significant.

## Abbreviations

AMPK: 5' AMP-activated protein kinase
Echo: echocardiography
ERK: Extracellular signal-regulated kinases
ET: endothelin
FDG: 2'-deoxy-2'-[^18^F]fluoro-D-glucose
FS: fractional shortening
GAPDH: Glyceraldehyde 3-phosphate dehydrogenase
HFpEF: heart failure with preserved ejection fraction
IVS: interventricular septum
LVEJ: left ventricular ejection fraction
LVID: left ventricular internal diameter
LVPW: left ventricular posterior wall thickness
MAPK: Mitogen-Activated Protein Kinases
PET: positron emission tomography
ROS: reactive oxygen species
SBP: systolic blood pressure
SUV: standard uptake values
TG2: transglutaminase 2
VOI: volume of interest
