# Supplementary material for: Sunitinib-induced cardiac hypertrophy and the endothelin axis
Source: Theranostics. 2021 Feb 6;11(8):3830–8. doi: 10.7150/thno.49837 (PMC7914356; doi:10.7150/thno.49837)
Supplement: Supplementary file 1 — Supplementary figures and tables. [file thnov11p3830s1.pdf]

Supplementary Material

Tumor growth

The tumor volume was measured by caliper and calculated following the formula: length x $(width)^2 / 2$. In the vehicle group tumor size had increased by 9 fold at week 3, while in the sunitinib-treated group tumor size was unchanged at week 3 and increased 3-4 fold at week 6.

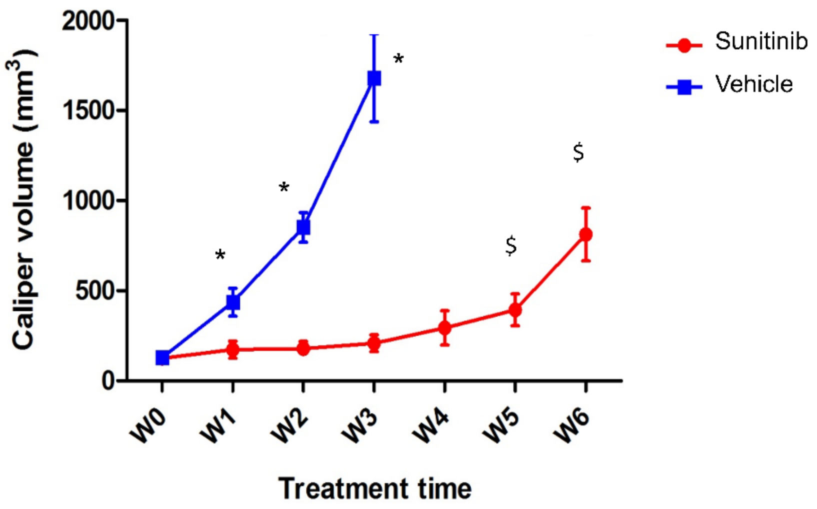

Figure S1. Effects of sunitinib on tumor and vessel growth of *Sdhb*$^{-/-}$ PGLs allografts. Quantification of tumor volume by calliper. $ $p < 0.05$ compared with W0, W1, W2 and W3 in sunitinib treated group (n = 8). Data are expressed as mean ± SEM. * $p < 0.05$ between the two groups using 2-way ANOVA. In the vehicle treated group (n = 8), all values at W1 and W3 are statistically different from baseline ($p < 0.05$).

Expression of MAPK (ERK) in sunitinib-treated myocardium

Proteomics and analysis with Ingenuity were performed as previously reported [1]. There was a trend towards activation of the MAP2K1 (ERK) pathway in the sunitinib-treated and the sunitinib+macitentan hearts, compared to vehicle **(Figure S2)**. MAP2K1 is involved in canonical pathways related to hypertrophy and oxidative stress **(Table S1).**

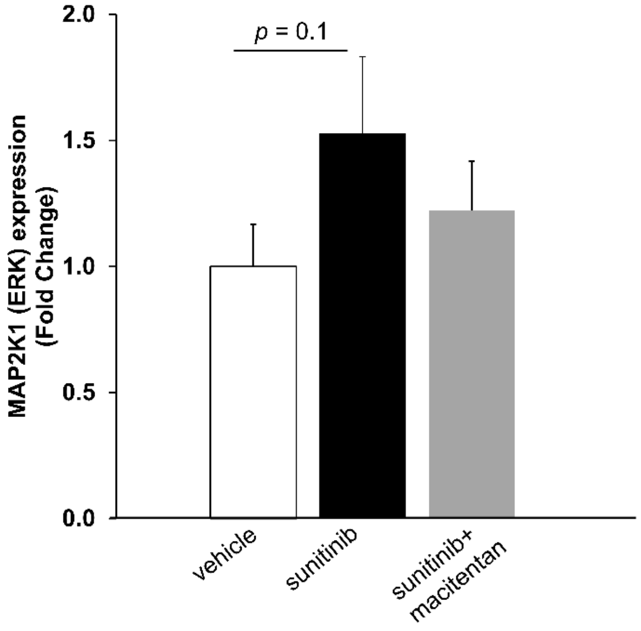

Figure S2. Sunitinib and expression of ERK. Relative expression of MAP2K1 (ERK). Data are expressed as mean ± SEM, n = 6 per group. ERK: Extracellular signal-regulated kinases; MAPK: Mitogen-Activated Protein Kinases.

		Sunitinib vs. vehicle		sunitinib+macitentan vs. vehicle group		Sunitinib vs. both sunitinib+macitentan & vehicle groups	
**Categories**	**Diseases or Functions Annotation**	**p-Value**	**Molecules**	**p-Value**	**Molecules**	**p-Value**	**Molecules**
Canonical pathways	Role of NFAT in Cardiac Hypertrophy	0.05	CAMK2D, RACK1, SLC8A1, **MAP2K1**	0.3	Calm1, CAMK2D, PDIA3, GNG12	n.s	-
Canonical pathways	Protein Kinase A Signaling	0.05	PYGM, CAMK2D, RHOA, RACK1, PPP1CB, PPP1R3A, CTNNB1, **MAP2K1**, TTN	0.01	YWHAQ, FLNB, CAMK2D, H3F3A/H3F3B, Calm1, YWHAB, PDIA3, ADD1, TIMM50, PPP1CB, PGP, APEX1, GNG12, HIST2H3C	n.s	-
Canonical pathways	NRF2-mediated Oxidative Stress Response	0.003	GSR, FTL, GSTM5, PPIB, **MAP2K1**, GSTP1, EPHX1, FTH1	0.008	GSTM1, STIP1, ACTB, NQO1, DNAJB6, ACTG1, CBR1, EPHX1, FTH1	n.s	-

Table S1. Changes in cardiac toxicity related pathways. Table showing the most significant canonical pathways that involve MAP2K1 (ERK) in mouse myocardium treated with sunitinib. n = 6 per group. MAPK: Mitogen-Activated Protein Kinases
